# Influence of Composition and Texture on In-Mouth Sodium Release and Saltiness During Consumption of Semi-Hard Cheeses

**DOI:** 10.3390/foods15091462

**Published:** 2026-04-22

**Authors:** Génica Lawrence, Chantal Septier, Fabrice Buchin, Christine Achilleos, Solange Buchin, Christian Salles

**Affiliations:** 1 Université des Antilles, Laboratoire COVACHIM-M2E (EA 3592), UFR SEN, F-97110 Pointe-à-Pitre Cedex, France; genica.lawrence@univ-antilles.fr; 2 Université Bourgogne Europe, Institut Agro, CNRS, INRAE, UMR CSGA, 21000 Dijon, France; chantal.septier@inrae.fr; 3 R & D Service, ENILEA Campus de Poligny, 16 Rue de Versailles, 39800 Poligny, France; fabrice.buchin@educagri.fr; 4 Université Bourgogne Europe, Institut Agro, INRAE, UMR PAM, 21000 Dijon, France; christine.achilleos@inrae.fr (C.A.); solange.buchin@inrae.fr (S.B.)

**Keywords:** in-mouth salt release, saltiness perception, texture, cheese, fat

## Abstract

Excessive sodium intake is a major dietary concern, leading to recommended reductions in several food categories, including cheese. This study aimed to evaluate how cheese composition and texture influence sodium release and perceived saltiness during consumption. Semi-hard cheeses (SHCs) with varying compositions were analyzed for chemical composition, rheological properties, and sensory attributes using quantitative descriptive analysis, temporal sodium release and saltiness intensity. Most compositional factors affected the sensory characteristics of SHCs and the dynamic perception of saltiness. In particular, salt level influenced not only the perceived intensity of saltiness but also bitterness, acidity, overall aromatic intensity, and numerous textural characteristics. The fat content also influenced texture perception and masked taste attributes. Moreover, both sodium release and saltiness perception decreased with increasing fat content. These findings highlight the importance of compositional and textural factors in modulating salt perception and provide useful insights for developing reduced-salt cheeses with acceptable sensory qualities.

## 1. Introduction

Excessive intake of sodium has undesirable effects on health and is the cause of pathologies such as hypertension and cancer, and indirectly leads to osteoporosis [1]. Several studies showed physiological links between salt intake and blood pressure [2,3,4].

Added sodium chloride is a major source of sodium in the diets of modern countries and public health organisms recommend to progressively reduce salt by 30% in foods identified as the main contributors to sodium intake [5,6], with a target of 5 g salt intake per day. However, recent research reported that additional, discretionary salt added by consumers during cooking, food preparation or during meals is also an important source of sodium intake in the diet [7]. In industrialized countries, 75–80% of dietary salt comes from the consumption of processed foods, 5–10% from the natural content of foods, and 10–15% is due to the discretionary addition of salt at the table and during food preparation [8]. The release of salt in the mouth and the perception of saltiness vary depending on these domestic salting practices [9,10]. Several strategies for sodium reduction in foods have been elaborated [11,12]. Nevertheless, no single universal strategy exists for salt reduction in food products; rather, approaches must be specifically tailored and optimized to preserve the functional and sensory properties of each target food. Among these foods, cheese is considered as a high sodium content food, despite important intra- and inter-variety differences between cheeses in salt content [13,14]. For example, cheese is estimated to contribute to about 4% of salt consumption in UK [14,15]. However, cheese also possesses several beneficial nutritional attributes, notably containing essential minerals such as calcium and potassium. In particular, cheese significantly contributes to protein, phosphorus and calcium supply in food [16].

The role of salt in cheese is multiple [13]: technological [17,18], preservative, organoleptic and safety. During ripening, it regulates enzyme activity, microbial growth and metabolism, thereby affecting proteolysis. From a sensory perspective, it contributes to saltiness perception, affects aroma release, decreases bitterness and indirectly influences flavour changes occurring during ripening [17,18]. Cheese with a higher salt content has less proteolysis during ripening and increased hardness and fracturability [13,18,19].

Flavour perception is a dynamic phenomenon depending on both temporal flavour compound release in the mouth during food oral processing and the oral physiology of the individual [20]. However, compared to volatile compounds, a limited number of studies have focused on the in-mouth release of non-volatile compounds. One early investigation reported that, with each chew, a greater amount of salt is released from firmer and drier Cheddar cheeses, which are characterized by low water content and a high salt-to-moisture ratio. Furthermore, the time required during mastication to reach the maximum salt release is directly related to the softness and creaminess of the Cheddar samples [21]. However, most subsequent studies have employed model matrices whose structure and composition are far less complex than those of real cheese to enable precise control of composition and to investigate the effects of matrix characteristics on temporal salt release and perceived saltiness.

The release of sodium and the perception of saltiness are influenced by food composition, including lipids, proteins, and water. However, the reported effects remain unclear and sometimes contradictory [22]. For example, in model processed cheeses obtained by the action of melting salts and heating, an increase in fat content has been associated with an overall decrease in sodium release and an increase in perceived saltiness [23,24,25]. In model dairy lipoprotein matrices obtained after renneting, compositional and textural variations modified the timing of sodium release and saltiness perception, but the effects depended on salt concentration [26]. This phenomenon may be explained by the binding of sodium ions to caseins [27,28], which modifies the cheese microstructure [29]. Furthermore, these observations were complemented by a meta-analysis highlighting the complexity of the relationships between matrix components, salt release, and saltiness perception [30]. Moreover, potential cross-modal perceptual interactions with saltiness perception may add complexity in the understanding of the direct relationships between this perception and the matrix composition [31,32,33].

In light of these observations on the complex relationships between salt perception and matrix structure, it is evident that the phenomena observed in cheese model matrices are poorly predictive of those that may occur in real cheeses, which undergo ripening and consequently at least partial transformation of proteins and lipids. Therefore, in a public health context addressing the reduction in salt content in cheese products, it appears essential to investigate the impact of salt reduction on salt perception during consumption, using real cheeses with controlled composition rather than simplified model foods which, to our knowledge, has never been done.

The objective of this study was to examine the effects of compositional modifications across a broad range of semi-hard cheeses (raclette-type) with controlled compositions on their sensory and rheological properties, in-mouth sodium chloride release, and temporal dynamics of saltiness perception.

## 2. Materials and Methods

### 2.1. Cheese Manufacture

**Figure 1:**
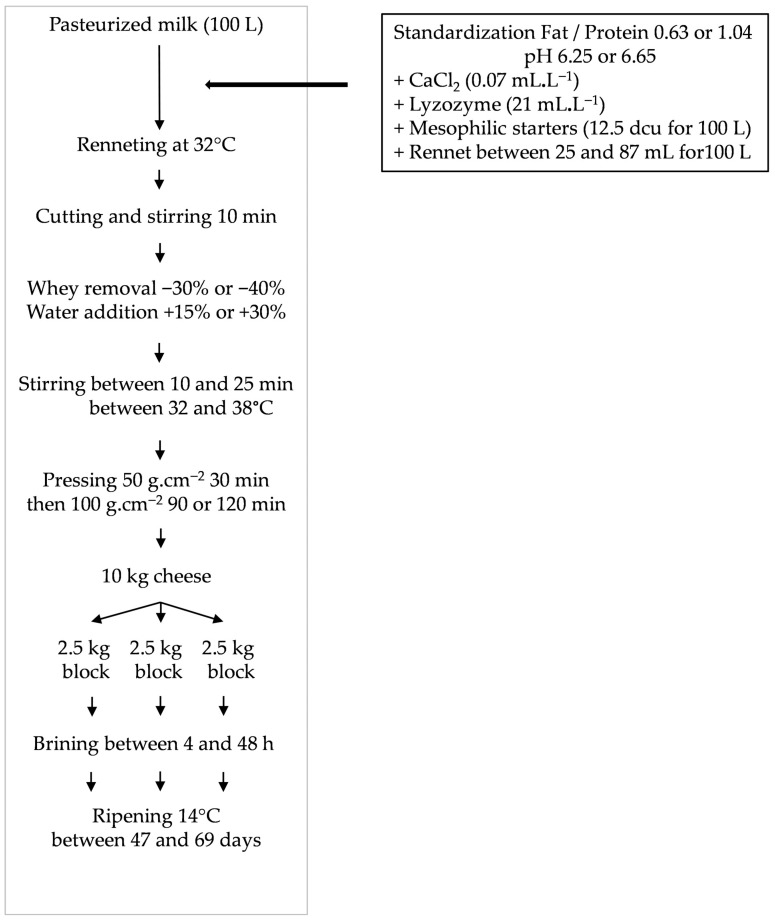
Technological scheme of semi-hard cheeses production.

Twenty-four semi-hard cheeses (SHCs) were made from pasteurized cow’s milk with variations in moisture in non-fat solids, fat in dry matter, calcium to non-fat dry matter ratio and salt to moisture ratio that covered the range encountered in commercial SHCs. The factor levels were as following: 60 and 64%, 40 and 50%, 2.5 and 2.8%, 2.0, 3.0 and 4.5% for moisture in non-fat solids, fat in dry matter, calcium to non-fat dry matter ratio and salt to moisture ratio, respectively. The 4 factors were combined following a complete experimental design to generate the maximum variability. Milk was standardized in fat to protein ratios at 0.63 or 1.04 *w*:*w*, inoculated with mesophilic starters MA014 (12.5 dcu for 100 L) (Danisco, Paris, France), and added with CaCl_2_ (0.07 mL.L^−1^ of 520 g.L^−1^ solution) and lysozyme (0.25 mL.L^−1^). The pH was adjusted to 6.65 with lactic acid or to 6.25 with glucono-delta-lactone (1.4 g.L^−1^), then rennet (Chymax+, CHR Hansen, St Germain les Arpajon, France) was added at 32 °C at 25 to 87 mL for 100 L of milk. After coagulation, the curd was cut, a part of the whey (30 to 40%) was removed, water (15 to 30%) was added, then the curd grains were stirred between 10 and 25 min at 32 to 38 °C. The grains were moulded and pressed 30 min at 50 g.cm^−2^, and then 120 min at 100 g.cm^−2.^ At unmoulding, each 10 kg cheese block was cut in 2.5 kg blocks (100 × 100 × 300 mm) that were brined between 4 and 48 h in saturated brine at 11 °C. After brining, blocks were left drying a few hours, then rubbed with natamycin, and lastly packed in a plastic bag under vacuum. They were ripened at 14 °C for 47 to 69 days, until the proteolysis extent, given by the ratio of water-soluble nitrogen to total nitrogen (WSN/TN), reached about 20% (Figure 1).

### 2.2. Rheological Measurements

The mechanical properties of the 24 SHCs were measured by uniaxial compression at a constant displacement rate [34]. Cheese samples were stored overnight at 15 °C to equilibrate to the measuring temperature. Cylindrical test samples (initial height-to-diameter ratio between 1.1 and 1.5) were taken from the cheese block and placed in a pill-box at 15 °C for 15 min before measurement to prevent dehydration and allow stress relaxation. Measurements were performed at 15 °C with a TA-XT2 Texture Analyser (Stable Micro Systems Ltd., Champlan, France). Samples were compressed at a constant crosshead speed along their main axis between two parallel plates. The displacement speed of the upper plate was set to 0.8 mm/s. Samples were compressed until fracture occurred, corresponding to a maximum deformation of 80% of the initial sample height. Four replicates were performed per sample. Using the recorded force and displacement data, engineering stress (σ = Ft/A_0_, Ft = recorded force and A_0_ = initial cross section) and Cauchy strain (ε = Δh/h_0_, Δh = displacement and h0 = initial sample height) were calculated. From these data, the modulus of deformability M_D_ (kPa), the fracture stress σf (kPa) and strain εf (dimensionless), and the work to fracture Wf (kJ/m^3^) were determined.

### 2.3. Sensory Profiling

The sensory attributes of SHCs were evaluated by a trained panel of fourteen graduate students in food science (18 to 20 years old), using a conventional sensory profiling [35]. The panellists evaluated the 24 SHCs by a sequential monadic profiling. For each sample, they were asked to rate texture (crumbly, firm, springy, covering, pasty, grainy) [36] and taste intensities (salty, sour, bitter) [37] on linear scales from 0 to 10 (0: none and 10: extremely strong). Six products were evaluated per session in a well-balanced order. Each product was evaluated in duplicate. Between each sample, consumers were asked to cleanse their mouth with bread without salt and mineral water. Each panellist participated to eight 1 h training sessions and eight 1 h measure sessions (2 sessions per week). All participants consented to participate to this study and were informed before starting it.

Cheese sample pieces were prepared in the morning before each afternoon tasting-session. The samples (2 rectangular pieces of 5 g) were served in a random order that was different for each session, following McFie tables [38]. The tests were conducted in an air-conditioned room (21 °C) in individual booths. Data acquisition was manually performed then compiled in Excel Microsoft^®^ software.

### 2.4. In Vivo Temporal Saltiness and Sodium Release Measurements

#### 2.4.1. Training of the Panel

Eighteen subjects (8 women, 10 men) were examined concerning salivation and masticatory performance. Among them, five subjects (3 women and 2 men—23 to 46 years old) were selected according to their oral parameters [39]. These subjects were different from those of previous sensory profiling. They were regular consumers of cheese products and reported to eat such food products at least once a week in a food habits questionnaire. Subjects participated in two 1 h sessions per week and were paid for their participation. They were introduced to the discontinue time-intensity evaluation of saltiness through a number of training sessions prior to the measure sessions. They were requested not to smoke or eat or drink flavoured foods at least one hour before the sensory session. Ethical considerations concerning this study were the same as reported above.

Sessions of sensorial analysis were all conducted in a tasting room with individual cabins; everyone had a personal computer, which permitted direct recording of tasting data using FIZZ-software (Version 2.46B; Biosystemes, Couternon, France). The room was air-conditioned at 21 °C. To train their perception for saltiness the subjects tasted four salt solutions with various concentrations of NaCl (0.25; 0.5; 1; 2 g/L) and rated the intensity on a scale from 0 to 10 compared to a reference solution with 2 g/L and pure water. Water represented the 0 level (not salty at all), whereas the reference was placed at 80% of the scale. Two replicates were performed. The last exercise for the subjects was to practice spitting and rating the taste intensity within 5 s.

#### 2.4.2. Measurement Sessions

The sessions were conducted three times for each of the five selected panellists, according two sequences classified as follows: 20, 40, 60, 80 s and 10, 30, 50, 110 s (5 panellists × 24 cheeses × 2 sequences × 3 replicates). During a same session, the sodium release and saltiness intensity were simultaneously recorded during the eating of SHCs. At the different times of the eating of 5 g sample, subjects were asked to spit one saliva sample (around 0.5 mL) in a 5 mL plastic tube of 13 mm diameter (Camlab Ltd., Cambridge, UK) and evaluated saltiness intensity on a scale anchored from 0 to 10 with regard to a reference which was one of the saltiest cheeses placed at 80% of the scale. Before the eating of each SHC sample, one saliva sample was collected (blank).

For each subject, two 1 h sessions per week were conducted at the same hour of the day for each session (A total of nine 1 h sessions). For each block, SHC samples were presented at 13 °C (±1 °C), in hermetically closed transparent coded cups, in random order and in red light. Between each sample, an interval of 90 s was imposed for mouth cleansing with apple, bread (without salt) and mineral water (Evian, France).

Sensory data acquisition was conducted with FIZZ software (Biosystems, Couternon, France). Saliva samples were stored at −20 °C until analysis.

#### 2.4.3. Analysis of Sodium Concentration in Saliva

Saliva samples were centrifuged for 5 min at 4 °C with 12,000 rpm, then diluted by factor 20, from 50 μL to 1 mL with 18 mOhm Milli-Q-water (Millipore, Bedford, MA, USA), membrane filtered (pore size 0.45 μm, C.I.L., Sainte-Foy-La-Grande, France) and analyzed through HPLC Ionic Chromatography [25] using a ICS2500 Dionex ion chromatographic system (Dionex, Voisins le Bretonneux, France). The cations were separated using an IonPac CG 12A-5 μm guard column and an IonPac CS12A 5 μm analytical column. Elution was achieved in an isocratic mode with 22 mM sulfuric acid (96% p.a, Carl Roth, Lauterbourg, France) at a flow rate of 0.5 mL/min. System control and data acquisition were accomplished using Chromeleon software (version 6.8). Quantification was performed with sodium standard solutions.

### 2.5. Data Analyses

Rheological properties of cheeses and sensory profiling data were analyzed using Analysis of Variance (ANOVA). A Principal Component Analysis (PCA) was used for analyzing the relationships between composition, rheological, sodium release and sensory parameters.

The effect of the composition on sodium release and saltiness perception was analyzed using MANOVA (Multivariate analysis of variance) and ANOVA. For each sodium release measurement sequence, the blank sample corresponding to time 0 was subtracted to all sodium measurements performed at the other times. The following parameters, extracted from the “time-intensity” curves, were considered: -slopes R1 and I1; increasing slope of the curve at the beginning of eating for sodium release and saltiness respectively, -Cmax and Imax; maximum concentration of sodium release and maximum saltiness reached, respectively, -TCmax and TImax; time to reach the Cmax and Imax, respectively, -slopeR2 and slopeI2; decreasing slope of the curve after reaching maximum concentration of sodium release and maximum saltiness intensity. These decreasing slopes represented the remanence of sodium in the mouth and saltiness persistence, respectively. The main effect of composition factors and subjects on sodium release and temporal saltiness were analyzed by ANOVA. When a significant effect (*p* < 0.05) was found by applying the ANOVA, the Student Newman-Keuls (SNK) test was used to compare the differences between the LS (Least Square) means. To analyze correlations between variables, Pearson’s correlation coefficients were calculated. The ANOVA were performed using a General Linear Model (GLM) procedure. The fixed factors were the 4 compositional factors (MNFS, F/DM, Ca/NFDM and S/M) and the factor panellists were used as random factors. All data analyses were carried out using STATGRAPHICS Centurion^®^ XV.I Software (Version 15.2, Sigma-Plus, France). The PCA was carried with Uniwin Plus (version 6.1, Sigma-Plus, France).

## 3. Results

### 3.1. Relationship Between Rheological, Sensory Properties and Composition of the Semi-Hard Cheeses

Table 1 (SHCs composition) shows that the setting of the four varying parameters fulfilled the objectives. More than the targeted values, the differences between paired cheeses for a given parameter are important. All the effects of composition factors on rheological and sensory properties of the SHCs are reported in Table 2. Several interactions between these factors were observed and their significance is reported in Table 3.

**Figure 2:**
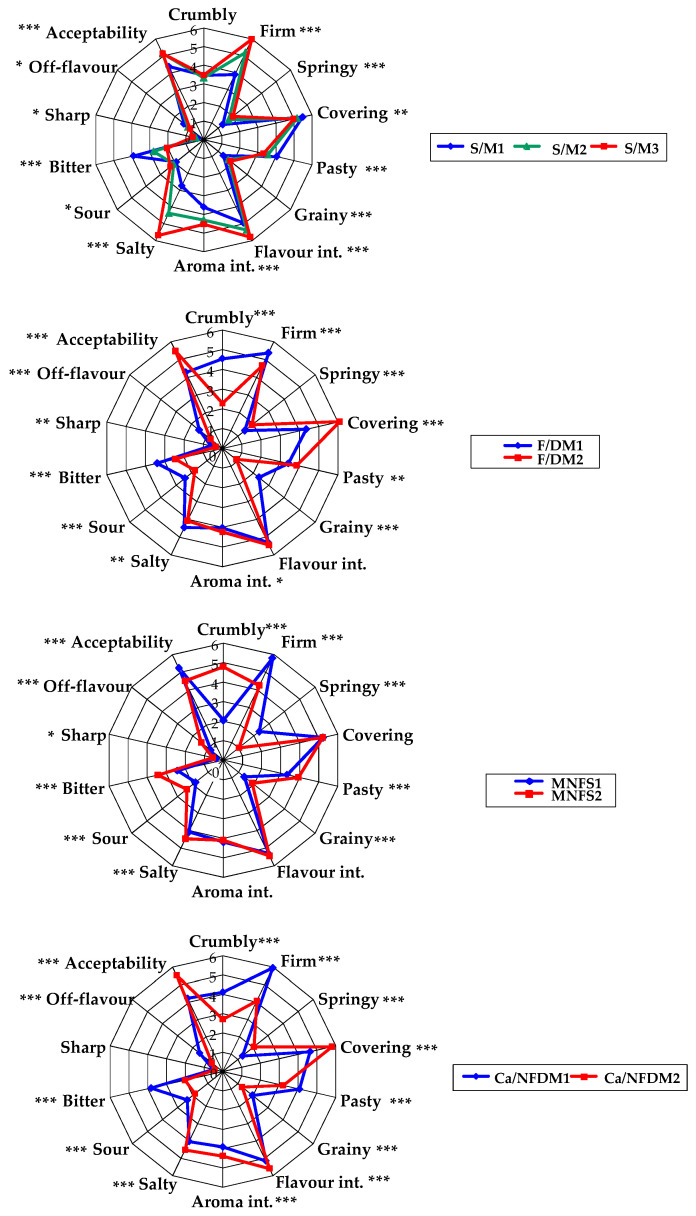
Spiderweb diagramme representing the results of the sensory profile of the cheeses according to their composition. *p*-value: *** *p* < 0.001; ** *p* < 0.01; * *p* < 0.05.

The results of the sensory profile can be visually observed in Figure 2.

Statistical analysis revealed the influence of cheese composition on rheology. The fracture stress (σf), i.e., the mechanical resistance to deformation of the cheese was chosen as the cheese hardness factor for this work. Almost all properties, except the calcium to non-fat dry matter ratio, showed a significant effect (*p* < 0.05) on fracture stress.

S/M: salt to moisture ratio; F/DM: fat in dry matter ratio; MNFS: moisture in non-fat solid: Ca/NFDM: calcium to non-fat dry matter ratio. 1: lower level; 2: higher level (3 for salt). MNFS1 and MNFS2: 60 and 63%; F/DM1 and F/DM2: 40 and 50%; Ca/NFDM1 and Ca/NFDM2: 2.5 and 2.8; S/M1, S/M2 and S/M3: 2.0%, 3.2% and 4.5%, respectively.

An increase in dry matter also led to more resistance towards mechanical deformation, which is shown by the significant correlation of these two parameters.

**Figure 3:**
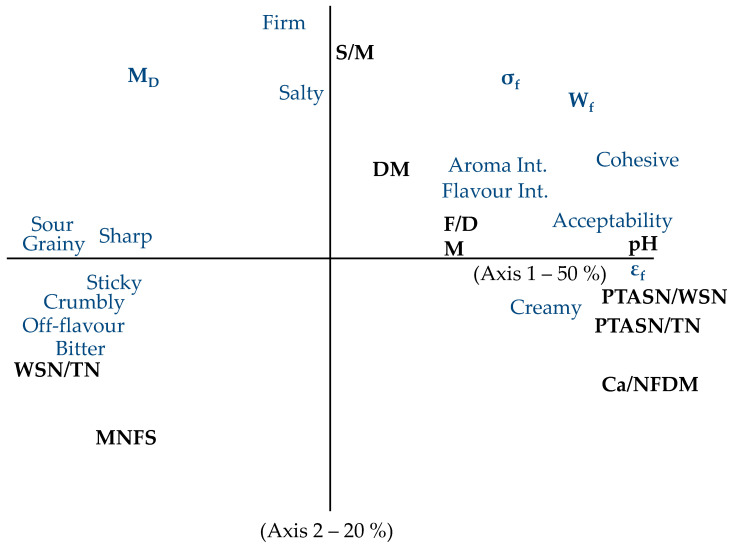
Principal component analysis plot for the average values of rheological data (modulus of deformability (M_D_), fracture stress (σf) and strain (εf), and the work to fracture (Wf)), analytical (pH, salt to moisture ratio (S/M), fat in dry matter ratio (F/DM), calcium to non-fat dry matter ratio (Ca/NFDM), water soluble nitrogen to total nitrogen ratio (WSN/TN), phosphotungstic acid soluble nitrogen to water soluble nitrogen ratio (PTASN/WSN), phosphotungstic acid soluble nitrogen to total nitrogen ratio (PTASN/TN), moisture in non-fat solids (MNFS) and sensory data obtained for each semi-hard cheese.

Figure 3 summarizes the main relationships observed between composition, rheology and sensory profile. Acceptability, phosphotungstic acid soluble nitrogen, and covering, strain at fracture were opposed—along axis 1—to sharp, bitter, water-soluble nitrogen which contains in particular bitter peptides, off-flavour, grainy, pasty. They are linked to pH and mineral content (Ca and P). A high pH was associated with high mineral content, dry matter, fat content and thin proteolysis (Phosphotungstic acid soluble nitrogen) and to a low water-soluble nitrogen value which gives cohesiveness to texture. The second axis was associated with moisture in non-fat solids value and salt to moisture ratio. The more moisture, the less firm and the more salt, the higher the firmness.

### 3.2. Influence of Composition on Sodium Release in Mouth and Salty Perception

For temporal sodium release and temporal saltiness perception, the increasing slope, the decreasing slope, the maximum signal intensity, and the time at which this maximum is reached were considered.

**Figure 4:**
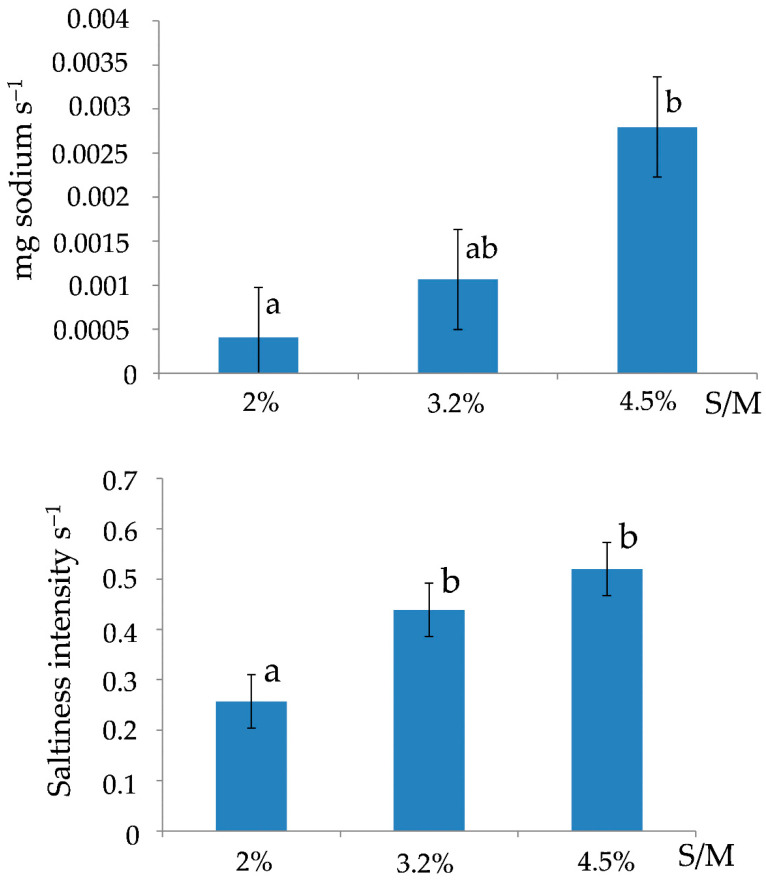
Release rate of sodium in saliva (SlopeR1) and speed of saltiness perception (SlopeI1) according to salt to moisture ratio (S/M), when eating semi-hard cheeses. Different letters indicate different statistical means according to the Student Newman-Keuls test (*p* < 0.05). Bars indicate the standard deviations (n = 15).

Increasing slope—A significant effect was observed only for salt concentration. Overall, both SlopeR1 (sodium release rate) and SlopeI1 (saltiness perception rate) increased with the salt to moisture ratio. No other significant effects of the cheese matrix components were observed for these parameters (Figure 4).

**Figure 5:**
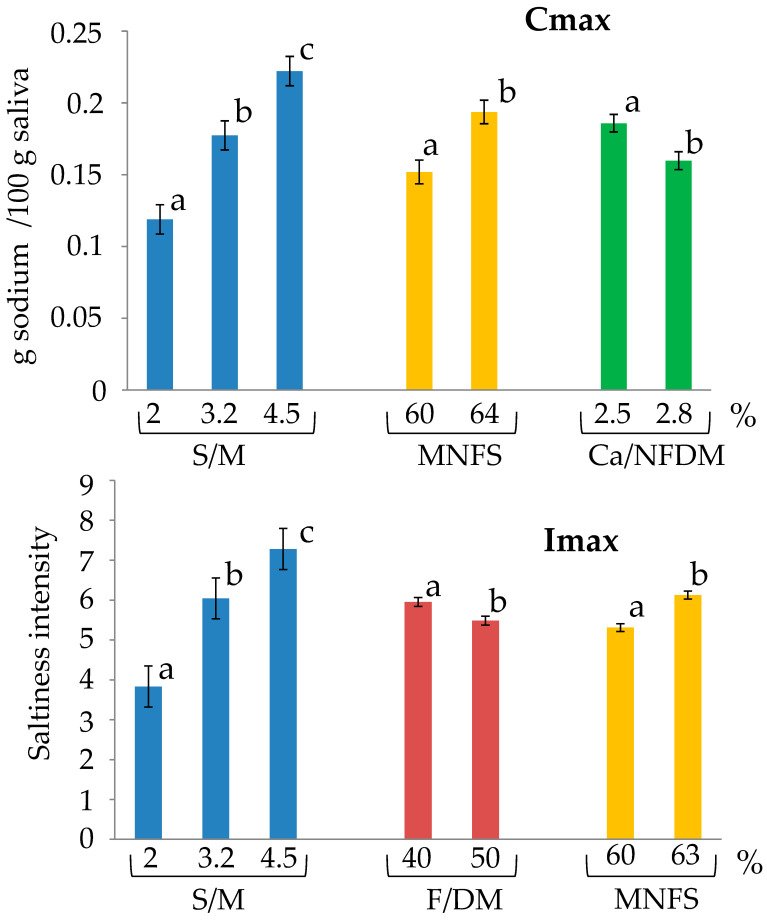
Maximum sodium concentration in saliva (Cmax) and maximum perceived saltiness intensity (Imax) according to salt to moisture ratio (S/M), fat in dry matter ratio (F/DM), moisture in non-fat solids (MNFS) and calcium to non-fat dry matter ratio (Ca/NFDM) when eating semi-hard cheeses. Different letters indicate different statistical means according to the Student Newman-Keuls test (*p* < 0.05). Bars indicate the standard deviations (n = 15).

**Figure 6:**
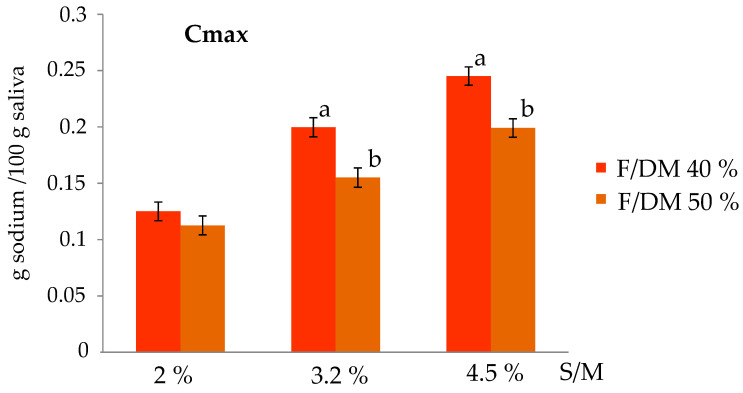
Maximum sodium concentration released in saliva (Cmax) when eating semi-hard cheeses according to salt to moisture ratio (S/M) for two fat in dry matter ratios (F/DM). Different letters indicate different statistical means according to the Student Newman-Keuls test (*p* < 0.05). Bars indicate the standard deviations (n = 15).

Maximum signal intensity—As expected, both Cmax (maximum sodium concentration in saliva) and Imax (maximum perceived saltiness intensity) increased with increasing salt to moisture ratio. Moreover, Imax decreased as the fat in dry matter ratio increased. Higher values of Cmax and Imax were observed when the moisture in non-fat solid was higher, whereas Cmax decreased when the calcium to non-fat dry matter ratio was higher (Figure 5). For Cmax, the fat in dry matter ratio effect was significant only at medium and high salt to moisture ratios, where higher fat in dry matter ratio corresponded to higher Cmax values. No comparable trend was observed for Imax (Figure 6). Only minor effects on Tmax were noted for both sodium release and saltiness perception.

**Figure 7:**
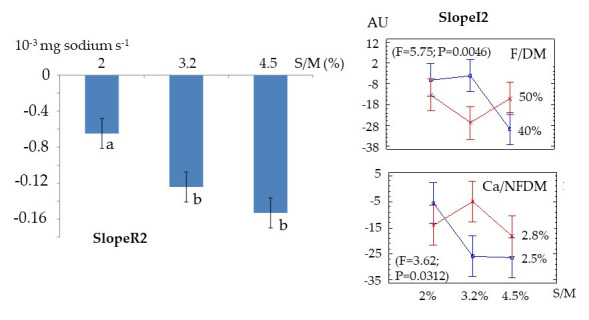
Persistence of sodium in saliva (SlopeR2) according to salt/moisture ratio and persistence of saltiness perception (SlopeI2) according to salt to moisture ratio (S/M), fat in dry matter ratio (F/DM) and calcium to non-fat dry matter ratio (Ca/NFDM) when eating semi-hard cheeses. Different letters indicate different statistical means according to the Student Newman-Keuls test (*p* < 0.05). Bars indicate the standard deviations (n = 15).

Decreasing slopes—SlopeR2 and SlopeI2—represent, respectively, the persistence of sodium in the mouth and of saltiness perception. A significant effect was found only for low and medium salt concentrations on SlopeR2: the rate of sodium decrease in saliva was lower at the lowest salt level. For SlopeI2, a significant effect was observed only at medium salt concentration, where saltiness decreased more rapidly with higher fat in dry matter ratio and lower calcium to non-fat dry matter ratio values (Figure 7).

## 4. Discussion

The results obtained from the analysis of texture perception and rheological properties of the SHCs were generally consistent and suggested that the rheological behaviour of SHCs could serve as a good predictor of their textural perception properties. Through a carefully controlled experimental design, SHCs with varying compositions were produced. These different SHCs exhibited mechanical, textural, and flavour characteristics that were, in some cases, interrelated.

A significant influence of cheese composition was observed on most rheological parameters. For instance, nearly all compositional variables, except the calcium to non-fat dry matter ratio, had a significant effect on fracture stress. A higher salt to moisture ratio was associated with firmer cheese, which is consistent with previous research [40]. This result can be explained by differences in the degree of casein hydration and aggregation influenced by varying salt concentrations [13,17]. Furthermore, the extent of proteolysis may also contribute to this observation, as low-salt or unsalted Cheddar cheeses have been shown to exhibit more extensive proteolysis than salted ones, resulting in reduced firmness [13,41]. This increased proteolysis has also been associated with greater bitterness. This phenomenon, which has been extensively documented, can be attributed to a more favourable environment for microbiological growth resulting from the higher moisture content. At lower salt concentrations, increased water activity provides more suitable conditions for microbial and enzymatic activities [42,43], and also promotes protein hydration, leading to a softer texture [17,41]. However, contradictory findings have been reported for certain cheeses such as Prado [44] and Cantal [45] where proteolysis was not affected by salt reduction [42].

The salt to moisture ratio also strongly influenced texture perception characteristics. Regarding SHCs texture, a higher salt content resulted in a firmer and grainier, but less cohesive and pasty structure, which is quite consistent with the rheological properties of SHCs although the numerous interactions between compositional factors complicate the interpretation, an observation consistent with previous studies [13,17]. Interestingly, an opposite trend was observed in model processed cheeses obtained by the action of melting salts and heat [23,24] and in model dairy lipoprotein matrices obtained after renneting [26], where an increase in springiness with a higher salt to moisture ratio was reported. These contrasting findings suggest that the impact of salt on textural perception was highly dependent on the matrix composition and structure, likely due to pronounced differences in microstructure and physicochemical interactions between sodium ions and the matrix components.

The salt to moisture ratio influenced also the flavour characteristics. As expected, an increase in the salt to moisture ratio led to a higher perception of saltiness. However, when saltiness intensity decreased, bitterness perception—strongly correlated with low-molecular-weight nitrogen fractions (mainly hydrophobic small peptides)—increased, mainly due to the negative effect of salt on microorganisms and proteolytic activities as mentioned above. However, we cannot exclude cross-modal interactions between bitter and salty taste modalities as in low-salt SHCs, bitterness intensity could be enhanced as a result of the reduced masking effect associated with lower saltiness perception [46]. This phenomenon may explain the higher overall acceptability of SHCs at higher salt concentrations, where saltiness perception is maximal and bitterness minimal. Sharpness and sourness followed the same trend as saltiness perception, which can be attributed to these sensations being flavour components associated with elevated salt levels.

As anticipated, fat content exerted a significant influence on texture perception, by enhancing the perception of coating, springiness, and pastiness, while reducing firmness, graininess, and crumbliness. The decrease in firmness associated with increased fat levels has been extensively documented [47,48,49,50]. Interestingly, the intensity of taste attributes such as sourness, saltiness, bitterness, and sharpness declined at the highest fat content. This phenomenon may be attributed to a masking effect of fat on these sensory perceptions. Notably, fat appeared to attenuate off-flavours and enhance overall acceptability.

Moisture in non-fat solids also influenced texture perception in a consistent manner. Water content is a major driver for firmness perception and related springiness (positively), pasty and crumbly sensations (negatively). The perceived intensities of bitterness, sourness, saltiness, and sharpness increased with higher moisture in non-fat solids value. This could be attributed to the high-water solubility of the compounds responsible for these perceptions, whose dissolution in saliva is facilitated, thereby enabling easier interaction with taste receptors. In addition, a higher water content can favour microbial proteolysis and the release of bitter peptides. The cheese samples with higher moisture content, characterized by greater bitterness were also less appreciated with higher off-flavour intensities. This underlines the role of bitterness in the acceptability of foods. The calcium to non-fat dry matter ratio, which in this context is linked to SHCs pH, naturally influenced textural attributes. Regarding flavour perception, SHC samples with lower acidity were perceived as less bitter and less sour but saltier. A similar trend was observed for these three taste attributes and overall acceptability as for variations in salt concentration, although the pattern differed for texture attributes. These results again suggest that the overall acceptability of SHCs in the context of the present study, was primarily driven by taste, in particular bitterness extent, rather than texture characteristics.

Concerning the sodium release profiles, the results were consistent with the sensory profiles. Indeed, the perceived saltiness assessed using the profiling method was highly correlated with Imax (r = +0.94, *p* < 0.001), as well as with the initial perception slopeI1 (r = +0.88, *p* < 0.001), the maximum sodium concentration in the mouth Cmax (r = +0.80, *p* < 0.001), the initial release rate slopeR1 (r = +0.74, *p* < 0.001), and the decreasing slopeR2 (r = −0.58, *p* < 0.01). Similarly, the temporal perception of saltiness was strongly related to the temporal pattern of sodium release in saliva during consumption, as exemplified by the strong correlation between Imax and Cmax (r = +0.85, *p* < 0.001). Regarding the overall effect of texture, a less cohesive structure appeared to promote a higher sodium release, although this had a minimal impact on perceived saltiness. While the influence of salt content on both temporal release and perception parameters was evident, the effects of other components of the cheese matrix on temporal sodium release and saltiness perception were more complex. The moisture content affected both the maximum sodium concentration in the mouth and the maximum saltiness intensity in a similar manner: the higher the moisture content, the greater the intensity.

The lower calcium to non-fat dry matter ratio allowed a higher maximum sodium concentration in the mouth, but without any effect on the maximum saltiness intensity. This may be due to the relatively small difference in sodium release compared to samples with higher calcium to non-fat dry matter ratio. The effect of fat content was rather intriguing. No influence of fat in dry matter ratio was observed on the maximum sodium concentration released in saliva. However, the maximum saltiness intensity was higher in samples with lower fat in dry matter ratio. In this case, sodium release appeared to be decoupled from saltiness perception, as already reported [25]. A perceptual interaction between fat and saltiness perception may account for this phenomenon [33]; however, the underlying mechanism appears to be complex and remains poorly understood. Nevertheless, when considering each salt level separately, an effect of fat on sodium release was observed at the highest salt concentration, while no changes in perceived saltiness were reported regardless of the salt concentration. This aspect requires further investigation to be fully elucidated.

The decreasing phase represented the residual presence of sodium in the mouth (SlopeR2) and the persistence of saltiness perception (SlopeI2). A higher salt to moisture ratio in the SHCs was associated with a faster elimination of salt from the mouth, which appeared relevant. However, no significant effect was observed for SlopeR2, while the effects on the persistence of saltiness were more complex. Indeed, saltiness persistence seemed to vary with the salt to moisture ratio, but this variation appeared to be modulated by the fat in dry matter and calcium to non-fat dry matter ratios, which interacted with the salt to moisture ratio. The discrepancies between the release of the stimulus and its perception could be attributed to differences in the underlying mechanisms, particularly to perceptual interactions between saltiness and texture perception. Nevertheless, this remains a hypothesis that requires further verification.

## 5. Conclusions

Variations in SHCs composition (fat in dry matter ratio, salt to moisture ratio, calcium to non-fat dry matter ratio, moisture in non-fat solids) influenced the structural, textural, and sensory properties of the product, including flavour perception, sodium release, and temporal saltiness perception. In particular, decreasing the salt to moisture ratio resulted in lower perceived saltiness and higher bitterness intensity and fat was found to mask taste attributes such as sourness, saltiness, and bitterness but to enhance overall acceptability. Interestingly, an increase in the salt to moisture ratio was associated with greater firmness, whereas the opposite trend has been reported for several types of dairy model cheeses made without the ripening stage. This finding suggests a major role of matrix composition and structure in the observed phenomenon, likely due to process-related differences—especially during ripening—that affect both protein and fat content and their structural organization. This also shows the limitations of using cheese models whose matrix structure is not very representative of real cheeses for studies related to the perception of flavour and texture.

In the temporal analysis, similar tendencies were observed as in the non-dynamic study; however, the compositional parameters influencing salt release and the temporal perception of saltiness differed. For instance, fat in the dry matter ratio affected the intensity of perceived saltiness but not sodium release, while the calcium to non-fat dry matter ratio showed the opposite pattern. This supports the hypothesis that distinct mechanisms, yet to be elucidated, underlie these two phenomena.

Substantial salt reduction in cheese products is not easy but our findings suggest it is possible without compromising acceptability [51] by adjusting salt concentration in combination with process parameters and matrix composition. Achieving this goal, however, requires a thorough understanding of the factors governing the perception of saltiness which is highly complex and still not well understood [52,53].

## Figures and Tables

**Table 1:** Composition of the cheeses after ripening: targeted values and recorded values (mean ± standard deviation when n = 3).

Targeted Values (%)								
MNFS	60	60	60	60	64	64	64	64
F/DM	40	40	50	50	40	40	50	50
Ca/NFDM	2.8	2.5	2.8	2.5	2.8	2.5	2.8	2.5
Recorded values (%) (n = 3)								
MNFS	60 ± 1	61 ± 1	60 ± 1	60 ± 1	64 ± 2	63 ± 1	65 ± 2	64 ± 1
F/DM	38.5 ± 0.7	38.5 ± 0.7	50.3 ± 0.1	43.7 ± 0.8	37.5 ± 0.4	38.7 ± 0.6	50.1 ± 0.6	50.3 ± 0.4
Ca/NFDM	2.69 ± 0.07	2.34 ± 0.07	2.73 ± 0.06	2.56 ± 0.08	2.48 ± 0.03	2.33 ± 0.09	2.59 ± 0.08	2.35 ± 0.04
WSN/TN (target: 20)	19.5 ± 1.0	21.4 ± 0.7	19.8 ± 1.1	20.4 ± 0.0	22.5 ± 0.5	22.3 ± 0.5	21.5 ± 1.5	22.4 ± 0.3
Recorded values (%) (n = 1)								
S/M1 (target: 2.0)	1.88	1.86	1.90	1.72	2.22	1.78	1.91	1.84
S/M2 (target: 3.0)	3.40	3.30	3.26	3.13	3.24	3.17	3.30	3.20
S/M3 (target: 4.5)	4.84	4.61	4.55	4.62	4.86	4.47	5.02	4.23

F/DM: fat in dry matter ratio; MNFS: moisture in non-fat solid; Ca/NFDM: calcium to non-fat dry matter ratio; S/M: salt to moisture ratio; WSN/TN: water-soluble nitrogen to total nitrogen ratio.

**Table 2:** LS (Least Square) of mean (±standard error) and fixed ANOVA model of rheological parameters and sensory attributes (texture and salty perception) for each composition factor (dry matter content, fat/DM ratio, Ca/NFDM and salt levels) of the semi-hard cheeses.

	S/M1	S/M2	S/M3	*p*	F/DM1	F/DM2	*p*	MNFS1	MNFS2	*p*	Ca/NFDM1	Ca/NFDM2	*p*
M_D_	237 c ± 92	369 b ± 127	461 a ± 189	***	400 a ± 149	311 b ± 173	**	372 ± 177	339 ± 157	ns	461 a ± 137	250 b ± 117	***
εf	0.43 ± 0.15	0.39 ± 0.12	0.40 ± 0.13	+	0.34 b ± 0.08	0.47 a ± 0.14	***	0.46 a ± 0.12	0.35 b ± 0.11	***	0.34 b ± 0.06	0.47 a ± 0.14	***
σf	98 b ± 39	129 a ± 46	149 a ± 44	***	119 b ± 39	131 a ± 54	+	158 a ± 38	92 b ± 26	***	128 a ± 37	123 b ± 56	ns
Wf	21 c ± 11	27 b ± 14	32 a ± 16	***	22 b ± 9	31 a ± 16	***	36 a ± 13	17 b ± 5	***	25 b ± 10	28 a ± 17	*
Crumbly	3.45 ± 3.07	3.33 ± 2.94	3.47 ± 2.89	ns	4.55 a ± 2.82	2.28 b ± 2.66	***	2.04 b ± 2.25	4.79 a ± 2.96	***	4.12 a ± 2.90	2.71 b ± 2.87	***
Firm	3.88 c ± 1.85	5.20 b ± 2.10	5.97 a ± 2.13	***	5.37 a ± 1.99	4.66 b ± 2.35	***	5.79 a ± 2.07	4.24 b ± 2.07	***	5.96 a ± 1.95	4.07 b ± 2.04	***
Cohesive	1.31 b ± 1.83	1.73 a ± 2.20	2.01 a ± 2.28	***	1.45 b ± 1.79	1.92 a ± 2.40	***	2.35 a ± 2.38	1.02 b ± 1.59	***	1.30 b ± 1.81	2.07 a ± 2.35	***
Creamy	5.49 a ± 2.10	5.18 b ± 2.07	4.99 b ± 2.04	***	4.35 b ± 1.86	6.09 a ± 1.92	***	5.23 ± 1.98	5.22 ± 2.17	ns	4.64 b ± 2.02	5.80 a ± 1.97	***
Sticky	4.05 a ± 2.31	3.56 b ± 2.20	3.32 b ± 2.23	***	3.43 b ± 2.31	3.86 a ± 2.20	**	3.35 b ± 2.17	3.94 a ± 2.31	***	4.08 a ± 2.16	3.21 b ± 2.28	***
Grainy	1.35 b ± 1.55	1.69 a ± 1.88	1.84 a ± 1.80	***	2.36 a ± 1.93	0.90 ± 1.19	***	1.37 b ± 1.52	1.89 a ± 1.94	***	1.98 a ± 1.84	1.28 b ± 1.61	***
Salty	2.75 c ± 1.60	4.37 b ± 1.81	5.69 a ± 1.92	***	4.45 a ± 2.24	4.08 b ± 2.04	***	4.07 b ± 2.03	4.46 a ± 2.25	***	4.02 b ± 2.11	4.51 a ± 2.16	***

Different letters indicate significant differences between LS means (SNK test); *p*-value: *** *p* < 0.001; ** *p* < 0.01; * *p* < 0.05; + *p* < 0.1 (tendency); ns: Not significant. Wf: Work to fracture; σf: Stress at fracture (mechanical resistance); M_D_: Modulus of deformability (elasticity) and εf: Strain at fracture (cohesiveness). S: salt; M: Moisture; F: fat; DM: Dry matter; MNFS: Moisture in non-fat solid; Ca: Calcium; NFDM: Non-fat dry matter; MNFS1 and MNFS2: 60 and 63%; F/DM1 and F/DM2: 40 and 50%; Ca/NFDM1 and Ca/NFDM2: 2.5 and 2.8; S/M1, S/M2 and S/M3: 2.0%, 3.2% and 4.5%, respectively.

**Table 3:** Single interaction significances from fixed ANOVA model (see Table 1) of rheological parameters and sensory attributes (texture and salty perception) for each composition factor of the semi-hard cheeses.

Interactions	S * F/DM	S * MNFS	S * Ca/NFDM	DM * F/DM	Ca/NFDM * F/DM	Ca/NFDM * MNFS
M_D_					*	
εf	+		*		***	*
σf				**		*
Wf		*	*	***	**	**
Crumbly		**	***	***	***	*
Firm	*	*		***	***	
Cohesive			*			***
Creamy			+	***	***	
Sticky						**
Grainy				***		
Salty		*		+	*	**

*p*-value: *** *p* < 0.001; ** *p* < 0.01; * *p* < 0.05; + *p* < 0.1. Wf: Work to fracture; σf: Stress at fracture (mechanical resistance); M_D_: Modulus of deformability (elasticity) and εf: Strain at fracture (cohesiveness). S: salt; M: Moisture; F: fat; DM: Dry matter; MNFS: Moisture in non-fat solid; Ca: Calcium; NFDM: Non-fat dry matter; MNFS1 and MNFS2: 60 and 63%; F/DM1 and F/DM2: 40 and 50%; Ca/NFDM1 and Ca/NFDM2: 2.5 and 2.8; S/M1, S/M2 and S/M3: 2.0%, 3.2% and 4.5%, respectively.

## Data Availability

The original contributions presented in this study are included in the article. Further inquiries can be directed to the corresponding author.
